# WTP for a QALY and health states: More money for severer health states?

**DOI:** 10.1186/1478-7547-11-22

**Published:** 2013-09-01

**Authors:** Takeru Shiroiwa, Ataru Igarashi, Takashi Fukuda, Shunya Ikeda

**Affiliations:** 1 Center for Public Health Informatics, National Institute of Public Health, 2-3-6 Minami, Wako, Saitama 3510197, Japan; 2 Department of Drug Policy and Management, Graduate School of Pharmaceutical Sciences, The University of Tokyo, 7-3-1 Hongo, Bunkyo-ku, Tokyo 1130033, Japan; 3 Department of Pharmaceutical Sciences, School of Pharmacy, International University of Health and Welfare, 2600-1 Kitakanemaru, Otawara, Tochigi 3248501, Japan

**Keywords:** Quality-adjusted life years, Willingness-to-pay, Threshold, Cost-effectiveness analysis

## Abstract

**Background:**

In economic evaluation, cost per quality-adjusted life year (QALY) is generally used as an indicator for cost-effectiveness. Although JPY 5 million to 6 million (USD 60, 000 to 75,000) per QALY is frequently referred to as a threshold in Japan, do all QALYs have the same monetary value?

**Methods:**

To examine the relationship between severity of health status and monetary value of a QALY, we obtained willingness to pay (WTP) values for one additional QALY in eight patterns of health states. We randomly sampled approximately 2,400 respondents from an online panel. To avoid misunderstanding, we randomly allocated respondents to one of 16 questionnaires, with 250 responses expected for each pattern. After respondents were asked whether they wanted to purchase the treatment, double-bounded dichotomous choice method was used to obtain WTP values.

**Results:**

The results clearly show that the WTP per QALY is higher for worse health states than for better health states. The slope was about JPY −1 million per 0.1 utility score increase. The mean and median WTP values per QALY for 16 health states were JPY 5 million, consistent with our previous survey. For respondents who wanted to purchase the treatment, WTP values were significantly correlated with household income.

**Conclusion:**

This survey shows that QALY based on the EQ-5D does not necessarily have the same monetary value. The WTP per QALY should range from JPY 2 million (USD 20,000) to JPY 8 million (USD 80,000), corresponding to the severity of health states.

## Introduction

In economic evaluation, cost per quality-adjusted life year (QALY) is generally used as an indicator of cost-effectiveness [1,2]. Cost per QALY is calculated by dividing between-group differences in cost by differences in obtained QALY, and is a type of incremental cost-effectiveness ratio (ICER). The principal of QALY, “a QALY is a QALY is a QALY” [3], implies that all QALYs are of equal value. The question, however, is whether all QALYs have the same monetary values. For example, does QALY from cancer treatment have the same value as QALY from flu prevention, from which many people can recover without severe complications? Should the gained QALYs of children suffering from genetic diseases (for which they are not responsible) and those of smokers affected by lung cancer be treated equally? Faced by these issues, it is unlikely that many people accept the concept “a QALY is a QALY is a QALY”. Of course, QALY cannot fully reflect the preferences of people in terms of their health states [4]. Hence, although QALY is a useful indicator to improve the interpretation and comparability of cost-effectiveness analysis, other important factors need to be simultaneously considered when decision is made.

Cost-effectiveness of healthcare technologies is often discussed by comparing ICER with a threshold, i.e., ₤20,000-₤30,000 per QALY according to the National Institute for Health and Care Excellence (NICE) guidelines [5]. Usually, this threshold is not explicitly stratified by other factors, which are qualitatively considered at the time of appraisal or decision making. However, severity of health states and value of QALY are important issues. For example, in the UK, the introduction of value-based pricing is being planned. A paper of Department of Health said, “Under value-based pricing, there would be higher thresholds for diseases with a higher ‘Burden of Illness.’ The most important factors contributing to the measurement of ‘Burden of Illness’ would be the severity of the condition and the level of unmet need” [6]. In the Netherlands, it has been suggested that the threshold be adjusted from €10,000 to €80,000 per QALY in proportion to severity.

Shah [7] reviewed empirical studies on severity of illness and healthcare priority setting. The results mostly show that greater resources are allocated to patients with severer illnesses. Almost all studies measured public preferences by choice, ranking exercise, or the person trade-off; however, it is unclear how severity is weighed in QALY-based decision making. Nord et al. [8] suggested a method to convert utility score to social value considering severity weights obtained by the person trade-off. Unfortunately, however, this method is not used widely for decision making. Accordingly, we applied the willingness-to-pay (WTP) method to obtain the monetary value of a QALY.

Our previous study [9] investigated the WTP values for one additional QALY using double-bounded dichotomous choice (DBDC), a contingent valuation method (CVM), in six countries (Japan, Korea, Taiwan, UK, Australia, and the US). The relationship between health states and WTP per QALY was not evaluated. The EuroVAQ study measured WTP values per QALY in nine countries in Europe (the Netherlands, UK, France, Spain, Sweden, Norway, Denmark, Poland, and Hungary) [10-12], and the results suggested that the WTP per QALY for worse health state is higher than that for other states. More empirical studies are needed to evaluate the relationship between WTP and health states. This may contribute to improved decision-making and reflecting the preferences of a greater number of people.

In Japan, the Ministry of Health, Labour and Welfare (MHLW) has started a discussion on the introduction of economic evaluation for decision-making from 2012. Until now, economic evaluation has rarely been applied to Japanese healthcare system. Although Japan has not decided on how to use economic evaluation, its pros and cons and applicability are now being considered. Although JPY 5 million to 6 million (USD 50, 000 to 60,000) per QALY is frequently referred to as a threshold monetary value in Japan, some people doubt the application of this single threshold value. Therefore, we need to conduct empirical research to determine the monetary value of a QALY.

## Methods

### Survey structure

We surveyed WTP per QALY for various treatment and end-of-life scenarios. To determine whether severity of health status influences the preferences of people for the monetary value of a QALY, we obtained WTP values for one additional QALY for six health states (from severe to mild). The six health states were defined using EQ-5D descriptions: two mild states (State 1 = 11121 [Japanese EQ-5D utility score of 0.769] and State 2 = 11212 [0.750]), two moderate states (State 3 = 22122 [0.619], State 4 = 11323 [0.519]), and two severe states (State 5 = 23322 [0.386], State 6 = 21333 [0.335]). As this study was designed to compare Japan with other Asian countries, we used the health states with similar EQ-5D scores relative to other Asian countries (Korea and Thailand), excluding unrealistic descriptions (e.g., a patient is confined to bed, but has no problem with daily activities). In addition, WTP per QALY for end-of-life situations was also examined. As two patterns of end-of-life situations were considered, a total of eight patterns were included in the survey.

### Questionnaires

In the treatment scenario, respondents were asked to imagine they had an assumed health state as described based on the EQ-5D (the six states mentioned above). If they did not receive any treatment, they would live with the described health state for XX months. After XX months, they would recover full health. However, if they bought a newly developed treatment and received it, they would immediately recover full health. For each given assumed health state, the WTP value was measured by the respondents’ willingness to purchase the treatment.

We also specified the following conditions to each respondent to clarify the assumed situation; (a) because the treatment was not reimbursed by public health insurance, the full amount had to be paid to receive it; (b) loss of income due to the illness need not be considered (it is compensated by social security, etc.); and (c) payment for the treatment will influence the respondents’ household.

The number of months (XX), which was changed depending on the health state, was calculated to equal gained QALY (0.2 QALY and 0.4 QALY). For example, in the case that the utility score of the health state was 0.40, the number of months was 12 x 0.2/(1–0.4) = 4 months (0.2 QALY) and 12 x 0.4/(1–0.4) = 8 months (0.4 QALY). This also applied to end-of-life scenarios. In the end, 16 questionnaires (8 patterns x 2 QALY types) were constructed (Table 1). The QALY gain was determined by experts through discussions to reflect general treatment and to set realistic duration in each scenario. Given the feasibility of the study, only two levels of gained QALY were applied.

**Table 1:** 16 patterns of questionnaire

**No**	**Health state**	**EQ5D description**	**QALY**	**Period (months)**
1	Mild	11121	0.2	10
2			0.4	21
3		11212	0.2	10
4			0.4	20
5	Moderate	11323	0.2	5
6			0.4	10
7		22212	0.2	6
8			0.4	13
9	Severe	21333	0.2	4
10			0.4	7
11		23322	0.2	4
12			0.4	8
13	End-of-life	21333	0.2	7
14			0.4	14
15		Life threatening	0.2	2
16			0.4	5

“End-of-life scenario 1” reflected the assumption that respondent life expectancy was one month in health state 6 [EQ5D description: 21333]. A newly developed treatment could prolong life expectancy by seven months (0.2 QALY) or 14 months (0.4 QALY) in health state 6. “End-of-life scenario 2” reflected a situation similar to that in our previous survey [10]. Specific descriptions of health states were not provided. It was assumed that respondents faced a life-threatening situation and life expectancy was one month. Respondents were asked about their willingness to purchase a treatment that could prolong life expectancy by two months (0.2 QALY) or four months (0.4 QALY) in perfect health.

### Double-bounded dichotomous choice and bid value

In the present study, double-bound dichotomous choice (DBDC) was used to obtain WTP for one additional QALY. A bid value was shown and the respondents were asked whether they would pay the bidding value for the treatment. The CVM guidelines of the National Oceanic and Atmospheric Administration (NOAA) in the US recommend the use of “Yes” or “No” vote [13]. In the DBDC method, the question about willingness to purchase the treatment is asked twice.

In our survey, respondents were asked whether they wanted to purchase the treatment. Respondents who answered “Yes” were randomly shown the following six bid values (Table 2): JPY 0.2 million (USD 2,000, USD 1 = JPY 100, 5% of Japanese GDP per capita), JPY 0.5 million (USD 5,000, 10%), JPY 0.8 million (USD 8,000, 20%), JPY 1.6 million (USD 16,000, 40%), JPY 3.2 million (USD 32,000, 80%) and JPY 4.8 million (USD 48,000,120%). Respondents were asked whether they would pay for the new treatment. Depending on the first answer, bid values were changed and second-stage bid values were shown to the respondents. If respondents answered “Yes” to the first question, a higher bid value was shown in the second question and vice versa. The range of second-stage bid values was JPY 0.1—6 million (USD 1,000—60,000).

**Table 2:** Bid value of double bound dichotomous choice method

**Assignment**	First bid-value*	**First answer**	Second bid-value*
1	20 (5%)	No	10
		Yes	40
2	40 (10%)	No	20
		Yes	80
3	80 (20%)	No	40
		Yes	160
4	160 (40%)	No	80
		Yes	320
5	320 (80%)	No	160
		Yes	480
6	480 (120%)	No	320
		Yes	600

*JPY 10,000 (percentage of GDP per capita).

### Example of a questionnaire

A respondent was presented with the following DBDC question.

“The following description is for assumed health status A. Please imagine that you have this health status. If you do not receive any treatment, you will recover perfect health status B after you live for X months in health status A.

Now a new treatment has been developed. You can immediately recover perfect health status B if you receive the new treatment. However this treatment is not reimbursed by public health insurance. You will have to pay the full amount to receive it. Please respond to the questions after considering the following situations: (a) payment for the treatment will influence your household; (b) income loss due to the illness need not be considered (it will be compensated by other types of income, such as social security).”

Q1: Would you purchase the treatment by the smaller amount ? [Yes/No]

Q2: Now please assume that the treatment cost is JPY YYY. Would you purchase the treatment? [Yes/No]

Q3: Now please assume that the treatment cost is JPY ZZZ. Would you purchase the treatment? [Yes/No]

### Respondents

We randomly sampled approximately 2,400 respondents from an online panel. The panel, which is the largest in Japan, comprises 1.5 million people (INTAGE Inc.). In Japan, the Internet penetration rate averages about 80%, and almost all people under the age of 50 years have access to the Internet. We recruited respondents aged 20 to 69 years, who were then stratified by age and sex. This study was conducted in November 2011.To avoid misunderstanding, we randomly allocated respondents to one of 16 questionnaires, with 250 responses expected for each pattern.

### Statistical analysis

Using the DBDC method, individual WTP values cannot be obtained. From the responses, an acceptance curve, which shows the relationship between probability answering of a “Yes” response and bidding values, was generated. The mean WTP was obtained by calculating the area under the acceptance curve (integrating acceptance curve). The values were converted to WTP per QALY by multiplying by 5 (0.2 QALY) or 2.5 (0.4 QALY).

The acceptance curve can be generated using a parametric or nonparametric method. We primarily used the nonparametric Turnbull method [14] to obtain the conditional WTP values (WTP_c_) of respondents who had a WTP more than JPY 0. Letting the percentage of people who answered “Yes” to the first question be p_Y_, the mean WTP can be calculated as “WTP_c_ x p_Y_”.

To determine influential factors for WTP, parametric methods (logistic regression for the first question and Weibull regression for the WTP part) were also applied to all data (except for end-of-life scenario), including household income (continuous), education level (university or graduate school = 1, reference: high school or less), employment status (full time = 1, reference: others), marital status (married = 1, reference: not married), utility scores of the presented health states (continuous), and QALY (0.2 QALY or 0.4 QALY). Statistical software R 2.15.0 and SAS 9.2 were used.

## Results

A total of 2,283 respondents completed the questionnaire. Demographic characteristics are shown in Table 3. Sampling stratified by sex and age was successful. The average household income of respondents was approximately JPY 6.2 million (USD 62,000, USD 1 = JPY 100), although that of the average Japanese household, excluding elderly households, was JPY 6.1 million (USD 61,000) in 2009 [15]. The proportion of the Japanese population in each region in 2010 was 11.7% in Hokkaido/Tohoku, 33.1% in Kanto, 16.9% in Chubu, 17.7% in Kansai, 5.9% in Chugoku, 3.1% in Shikoku, and 11.5% in Kyushu [16]. Respondent demographics were similar to those of the Japanese general population. According to the Organization for Economic Co-operation and Development [17], data from 2009 indicated that 25% of the Japanese population attained a tertiary education (type A, college, or graduate). In our sample, 56% of respondents graduated from college or graduate school. The education level was somewhat higher in the study population.

**Table 3:** Demographic characteristics of respondents

	**N**	**Percentage**
Age		
20<= <30	444	19.5%
30<= <40	459	20.1%
40<= <50	449	19.7%
50<= <60	462	20.2%
60<=	469	20.5%
Sex		
Male	1160	50.8%
Female	1123	49.2%
Region		
Hokkaido/Tohoku	191	8.4%
Kanto	936	41.0%
Chubu	337	14.8%
Kansai	482	21.1%
Chugoku	95	4.2%
Shikoku	56	2.5%
Kyushu	186	8.2%
Household income (JPY 10,000)		
<100	71	3.1%
100<= <200	110	4.8%
200<= <400	560	24.5%
400<= <600	633	27.7%
600<= <1000	633	27.7%
1000<= <1500	209	9.2%
1500<= <2000	46	2.0%
<2000	21	0.9%
Employment		
Full-time worker	1019	44.6%
Part-time worker	339	14.9%
Self employment	178	7.8%
Homemaker	572	25.1%
Others (retirement…)	175	7.7%
Education		
University or graduate	1277	55.9%

**Figure 1:**
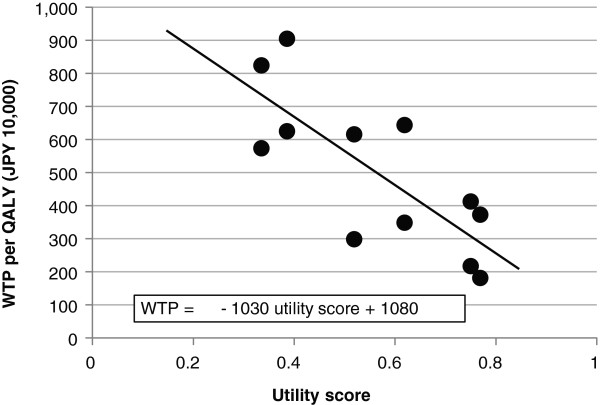
WTP for QALY and health states.

The mean WTP values per QALY from each questionnaire are shown in Table 4, and the relationship between WTP and utility score is presented in Figure 1. Figure 1 clearly shows that respondents were willing to pay more money for worse health states (less utility scores), with the regression line ranging from JPY 2 million (USD 20,000) to JPY 8 million (USD 80,000). The slope between WTP values and utility score was about JPY −1 million (USD 1,000) per 0.1 utility score increase in the treatment situation. The mean and median WTP values per QALY for 16 health sates were JPY 5 million (USD 50,000), consistent with our previous study.

**Table 4:** Estimate of WTP values

			**Q1**		
	**QALY**	**N**	**Yes**	**No**	WTP per QALY*
Mild					
state1: 11121	0.2	157	73 (46%)	84 (54%)	373
	0.4	139	61 (44%)	78 (56%)	181
state2: 11212	0.2	146	72 (49%)	74 (51%)	412
	0.4	139	73 (53%)	66 (47%)	217
Moderate					
state3: 11323	0.2	144	84 (58%)	60 (42%)	615
	0.4	134	84 (63%)	50 (37%)	298
state4: 22212	0.2	147	88 (60%)	59 (40%)	644
	0.4	134	90 (67%)	44 (33%)	349
Severe					
state5: 21333	0.2	149	93 (62%)	56 (38%)	824
	0.4	139	90 (65%)	49 (35%)	573
state6: 23322	0.2	141	96 (68%)	45 (32%)	905
	0.4	150	97 (65%)	53 (35%)	625
End-of-life					
state7: 21333	0.2	139	43 (31%)	96 (69%)	534
	0.4	142	67 (47%)	75 (53%)	524
state8: life threatening	0.2	132	42 (32%)	90 (68%)	537
	0.4	151	42 (28%)	106 (70%)	280
* JPY 10,000					

Results of multivariable analysis are shown in Table 5. Logistic analysis of the first question (respondents were asked whether they wanted to purchase the treatment) revealed that household income, education level, and marital status significantly affected the respondents’ willingness to purchase. Furthermore, lower utility scores were associated with a higher probability of a “Yes” response to the first question. For respondents who wanted to purchase the treatment, WTP values were significantly correlated with household income. The amount of gained QALY (0.2 QALY or 0.4 QALY) and utility scores of the presented health states significantly affected the WTP values.

**Table 5:** Relation between WTP values and demographic factors

	**Q1**		**WTP**	
	**Estimate**	**p-value**	**Estimate**	**p-value**
Household income (per JPY 1 million)	0.0623	<0.001	0.0474	0.005
Education level	0.2161	0.041	0.0400	0.744
Pattern of employment	0.2000	0.063	0.0832	0.514
Marriage status	0.3348	0.002	−0.1922	0.153
Utility score of presented health State	−1.7648	<0.001	−2.7035	<0.001
Scenario of QALY	0.0838	0.404	0.2381	0.044

Q1: Willing to pay more than JPY 1 for treatment.

WTP: Willing to pay the presented price in the questionnaire for treatment.

## Discussion

In this study, we examined the WTP values for one additional QALY and the relationship between WTP and health states. In agreement with our previous empirical study, the mean and median WTP values per QALY for 16 health state were JPY 5 million. Based on the results of regression analysis (Figure. 1), the WTP per QALY should range from JPY 2 million (USD 20,000) to JPY 8 million (USD 80,000), corresponding to the severity of health states. It is possible that the choice of gained QALY (0.2 or 0.4) influenced the WTP per QALY, and that it is easier for respondents to accept the payment for lower price treatment: these are limitations of our survey.

The following three interpretations are possible to deliberately state that WTP per QALY is correlated with the severity of health: (a) EQ-5D does not fully capture individual health state preferences; (b) WTP inadequately measures individual monetary value of a health state; and (c) the monetary value of a QALY differs depending on the severity of health states. From our empirical data, we cannot exclude either one of the three interpretations. For example, it is not deniable that scale of EQ-5D doesn’t have the same interval with individual preference for health states . However, we can conclude that at least in this study, WTP per EQ-5D-based QALY does not appear to be constant.

The principal “a QALY is a QALY is a QALY” is not necessarily supported by our results, when it comes to EQ-5D-based QALY. In the Netherlands, higher thresholds (from €10,000 to €80,000) are allowed for interventions aimed at increasingly severe illnesses [18]. Our results suggest that these types of decision making may reflect the preferences of people more appropriately. In the Netherlands, the suggested slope of the threshold is € 7,000 (JPY 0.9million, € 1 = JPY130) per 0.1 point severity increase. This value is similar to our slope of JPY 1 million (€8,000), although the unit of the denominator is different. Of course, from the empirical data, we cannot say anything about WTP per QALY out of the range of the utility score surveyed in this study.

If WTP is regarded as a factor for decision making based on cost-effectiveness, it may be possible to change the threshold based on the severity of health states to better reflect public preferences. Of course, other factors that influence various social values should be simultaneously considered. Some studies suggested introducing rules that guide QALY-based decision making, including the Fair innings rule [19], “Rule of Rescue” Egalitarian rule [8], and proportional shortfall [20]. These rules are distinct from the concept “a QALY is a QALY is a QALY.” However, these rules, as well as severity-weighed QALY, conflict with equity of QALY, which is applied to guidelines of economic evaluations in some countries [21,22]. A priority for severer patients may lead to discrimination of patients with a milder health state.

In the “end-of-life” guidance, NICE is allowed to use the utility scores of healthy people if the following conditions are met [23]: (a) life expectancy is less than 24 months; (b) life expectancy can be prolonged by 3 months; and (c) no alternative treatment is available. However, in our survey, the WTP in an end-of-life situation is not higher than that for a [21333] health state in treatment. Given that this result is exploratory and limited, the relationship between the end-of life situation and the WTP per QALY should be more carefully evaluated.

The EuroVAQ study applied the so-called “chained method,” in which respondents evaluate health states using standard gamble (SG) or both time trade-off (TTO) and WTP. According to the authors, the benefit of the EuroVAQ method is that utility score can be obtained from the same population in which WTPs are measured. The EuroVAQ study also considered risk scenarios, which measured WTP to avoid the possibility of the targeted health state in addition to time scenarios similar to those in our questionnaire. We think that risk scenario is important, and it will be considered in our future study.

Few studies have reported the measurement of WTP per QALY in general settings. In some studies, the WTP per QALY was calculated from the WTP for specific diseases. The WTP value per QALY, as determined by Gyrd-Hansen [24], was DKK 88,000. Using a method similar to that of Gyrd-Hansen, Bobinac et al. [25] also measured WTP per QALY and reported values ranging from €12,900 (based on VAS valuations) to €24,500 (based on the Dutch EuroQoL tariffs). Although the respondents were patients, King et al. [26] reported WTP per QALY ranging from USD 12,500 to USD 32,200. These results are lower than our estimated WTP per QALY. The WTP per QALY from the EuroVAQ study was different between the countries (Spain or Denmark was the highest and the Netherlands, France, and UK constituted the lowest group among 10 countries). The total means ranged from €20,000 to € 35,000. Although methods of existing studies differ, similar WTP values can be obtained from other studies.

QALY is a useful tool for valuing health states to improve the interpretation and comparability of cost-effectiveness analysis. Yet, if we reject the unitarism of QALY, a Pandora's box of conflicts – with regard to values and factors weighed in decision making – may be opened. Of course various factors in addition to cost per QALY are implicitly and qualitatively considered at present [27]. Surely, decision making in medicine should not be subject to people’s preferences alone; however, it is important to empirically clarify people’s preferences to improve the decision-making process and obtain social consensus. Our survey alone is not sufficient to this end, but it may contribute to this goal.

## Conclusion

In this study, we examined WTP values for one additional QALY and relation between WTP and health states. The mean and median WTP values for QALY of the 16 scenarios were around JPY 5 million. WTP per QALY for worse health states are higher than those for better states. Although the number of health states is not large in this survey, the slope is about JPY −1 million (USD 10,000) per 0.1 utility score increase. The results of the regression analysis suggested that the WTP per QALY should range from JPY 2 million (USD 25,000) to JPY 8 million (USD 100,000). Therefore, it may be possible to change the threshold based on the severity of health states to better reflect public preferences.

## Abbreviations

CVM: Contingent valuation method; EQ-5D: EuroQoL 5-dimension; DBDC: Double-bounded dichotomous choice; ICER: Incremental cost-effectiveness ratio; MHLW: Ministry of health, labour and welfare; QALY: Quality-adjusted life year; WTP: Willingness to pay.

## Competing interests

The authors declare that they have no competing interests.

## Authors’ contributions

TS completed the analysis and drafted the manuscript. AI, SI, TF appraised the quality of studies. All authors participated to design this study and approved the final manuscript.
